# Sleep Hygiene, Daytime Sleepiness, and Coping Mechanisms Amongst US Adults

**DOI:** 10.7759/cureus.45608

**Published:** 2023-09-20

**Authors:** Lindsey Yared, Kiran Rodrigues, Rohan Mangal, Thor S Stead, Latha Ganti

**Affiliations:** 1 Biology, Trinity Preparatory School, Winter Park, USA; 2 Biology and Medicine, Brown University, Providence, USA; 3 Medicine, University of Miami Miller School of Medicine, Miami, USA; 4 Medicine, The Warren Alpert Medical School of Brown University, Providence, USA; 5 Medical Sciences, The Warren Alpert Medical School of Brown University, Providence, USA; 6 Emergency Medicine and Neurology, University of Central Florida College of Medicine, Orlando, USA

**Keywords:** survey analysis, sleep, sleep problems, epworth sleepiness scale, sleep hygiene

## Abstract

Background

Excessive daytime sleepiness (EDS) can be measured by the Epworth Sleepiness Scale (ESS) and has been shown to be prevalent in the United States. This study aimed to survey the levels of EDS in US adults and explore factors related to sleep hygiene.

Methods

An anonymous web-based survey was conducted, polling the frequency of hypersomnia symptoms, sleep quality, and time spent in their occupation. Respondents were at least 21 years of age and United States residents. Participants reported information related to age, gender, education, income, and race in addition to their responses to the survey questions. All data were analyzed using JMP 16.0.

Results

In our cohort of 200 adults, the median age was 40, with an interquartile range of 34-50 years. 48% were female. More than half of the cohort (53%) had severe or higher-normal EDS. Race (p=0.112), sex (p=0.426), age (p=0.063) shift work/shift timing (p=0.260), and screen time before bed (p=0.785) were not statistically significant for excessive daytime sleepiness. However, the length of participants’ workdays (p=0.001) and their income levels (p=0.008) were found to be significantly associated with EDS. In other words, longer workdays and lower income levels were associated with an increased likelihood of falling asleep during inactive periods of the day.

Conclusion

Sleep deprivation and excessive daytime sleepiness are intertwined with sleep hygiene. This study highlights some of the possible issues that could lead to potential solutions.

## Introduction

Sleep is beneficial in many ways to the human body and is necessary for survival. Although its exact function is still unconfirmed, sleep has been demonstrated to be essential for learning and memory consolidation, energy conservation, and the removal of toxins from the brain [1]. Proper sleep has also been shown to protect against the incidence of depression and infectious diseases. In contrast, improper sleep can promote inflammatory pathways, influencing the progression of diseases like cancer and cardiovascular disease [2]. Poor sleep quality can affect someone’s daytime performance, including poor judgment and loss of concentration and energy [1]. For adults who are older than 25, it is recommended to get seven to nine hours of sleep daily to have optimal functioning and promote health [3].

Excessive daytime sleepiness (EDS) can be the result of a sleep disorder or an inadequate amount of sleep. Examples of sleep disorders include obstructive sleep apnea, restless leg syndrome, narcolepsy, insomnia, and parasomnias [1]. On the other hand, sleep can be interrupted or affected by many other factors, including chronic and acute illnesses, job shifts, substance abuse, screen time, children, and more. For example, excessive screen time usage before sleep can worsen sleep quality as the blue light emitted from devices can stop or alter the body’s ability to release melatonin [4]. Some of these interruptions may result in the development of sleep disorders like insomnia. In the United States, insomnia is the most prevalent sleep disorder [5]. Individuals dependent on substances are five times more likely to develop insomnia than the average adult [6]. Additionally, adults working early morning, rotating, or night shifts may also have abnormal sleep patterns that can potentially result in shift work disorders indicated through insomnia and EDS [7].

The development of EDS can be measured by the Epworth Sleepiness Scale (ESS). This scale is an eight-question survey that calculates how much daytime sleepiness someone has based on their answers to everyday scenarios. Participants can answer on a four-point scale per question, and scores are cumulatively tallied to give a score between 0 and 24, with different levels of daytime sleepiness indicated by the person’s score [8]. There are five different levels of daytime sleepiness, as indicated by the ranges of the cumulative sleep score. These levels are lower normal daytime sleepiness, higher normal daytime sleepiness, mild excessive daytime sleepiness, moderate excessive daytime sleepiness, and severe excessive daytime sleepiness. The test has demonstrated internal consistency through item and factor analysis, test-retest reliability, and one primary dimension of variance [9]. It has also demonstrated external validity. The ESS test has also been shown to demonstrate EDS the best among other sleep tests, like the maintenance of wakefulness test and the multiple sleep latency test [10].

This study aimed to survey 200 participants about sleep quality to better understand the incidence of poor sleep, sleep disorders, and factors potentially affecting sleep.

## Materials and methods

The study was conducted via an anonymous web-based survey in June 2022, using a survey research platform that uses organic sampling based on random device engagement. Through the platform’s integrated algorithms, single users on multiple accounts are excluded to ensure each response represents a unique individual participating in the study. The delivery of surveys to real consumers in targeted demographics occurs while they are organically engaged with the apps on their devices. While no personally identifiable information is collected, global information pertaining to age, gender, education, income, location (state), and race is obtained.

To be considered eligible for the study, participants were required to be 18 years of age or older and live in the United States. No further screening criteria were used, and the survey was administered in English. The study aimed to poll the frequency of hypersomnia symptoms, sleep quality, and time spent in respondents' occupations. The first eight survey questions asked participants about their level of sleepiness at various parts of the day, including periods of inactivity. These questions were based on the ESS, which is a validated subjective scale to measure daytime sleepiness in typical life situations. It is graded on a scale from 0 to 42. The following two questions asked participants about their regular sleeping and waking times and time spent viewing a screen, including a smartphone, television, and computer. The remaining survey questions were related to sleep medication use and the timing of work. This survey study did not use a power analysis (ad hoc) for a specific minimum sample size. All data collected from the survey were analyzed using JMP 16.0. The significance threshold was alpha = 0.05, and all confidence intervals were 95%. The study was deemed exempt by our institutional review board, study # 2023-423.

## Results

There were 200 survey respondents in the study, of whom 106 (53%) were male and 94 (47%) were female. Study participants had a median age of 40 years, an interquartile range of 33-50, and a range of 25-76 years. By background, 134 (67%) individuals identified as White/Caucasian, 27 (13.5%) as Black/African/African American, 16 (8%) as Hispanic/Latino, 4 (2%) as Asian, and 19 (9.5%) indicated other. Income levels in the cohort are represented as follows: 48 (24%) reporting earnings of under $25,000, 52 (26%) earning between $25,000 and $49,999, 27 (13.5%) between $50,000 and $74,999, 23 (11.5%) between $75,000 and $99,999, 20 (10%) between $100,000 and $124,999, 6 (3%) between $125,000 and $149,999, and 13 (6.5%) earning above $150,000.

Epworth Sleepiness Score

**Figure 1:**
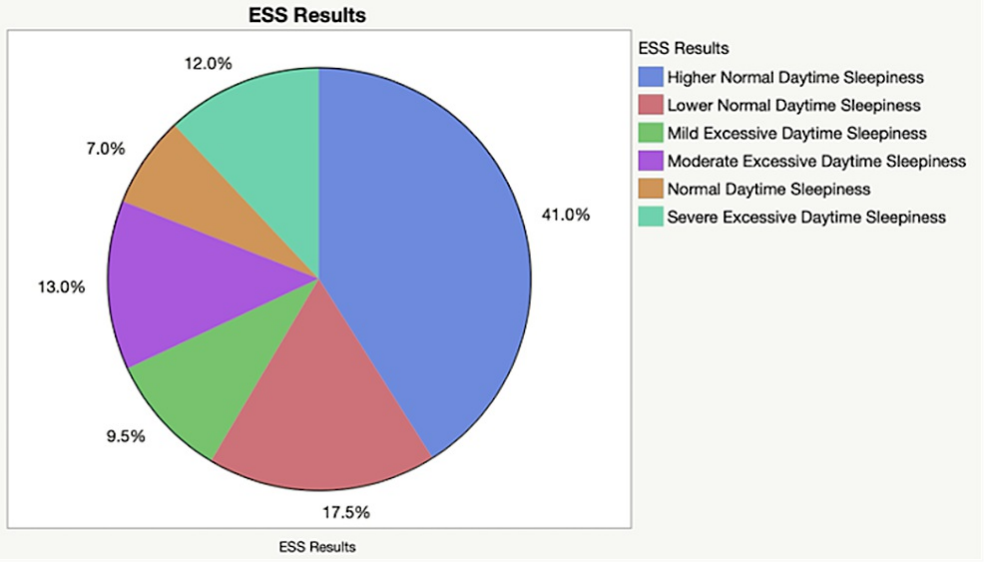
Summary of daytime sleepiness level in the cohort

The ESS is a score that ranges from 0 to 24. Scores are categorized as follows: 0-5: lower normal daytime sleepiness; 6-10: normal daytime sleepiness; 11-12: mild excessive daytime sleepiness; 13-15: moderate excessive daytime sleepiness; 16-24: severe excessive daytime sleepiness. The percentages of respondents' scores are depicted in (Table 1) and summarized in (Figure 1).

**Table 1:** Aggregate of responses to each of the Epworth Sleepiness Scale questions

ESS Question	Would never nod off	Slight chance of nodding off	Moderate chance of nodding off	High chance of nodding off
Sitting and reading	15%	38%	27%	18.5%
Watching TV	8.5%	40%	33.5%	17%
Sitting, inactive, in a public place (e.g., in a meeting, theater, or dinner event)	52.5%	29%	12%	5.5%
As a passenger in a car for an hour or more without stopping for a break	30%	32.5%	21%	15.5%
Lying down to rest when circumstances permit	3.5%	28.5%	37.5%	29.5%
Sitting and talking to someone	71%	16%	9%	3%
Sitting quietly after a meal without alcohol	37.5%	37.5%	16%	8.5%
In a car, while stopped for a few minutes in traffic or at a light	74.5%	13.5%	9.5%	1.5%

Amount of sleep and screen time

**Figure 2:**
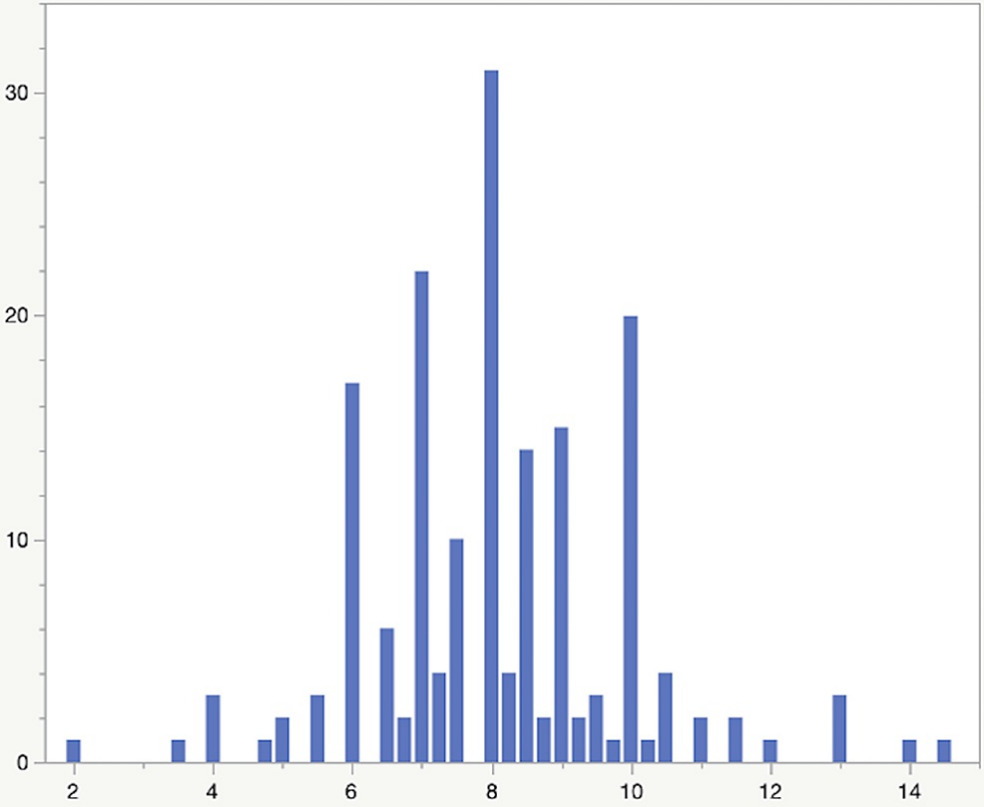
Number of hours respondents slept on average, per day

**Figure 3:**
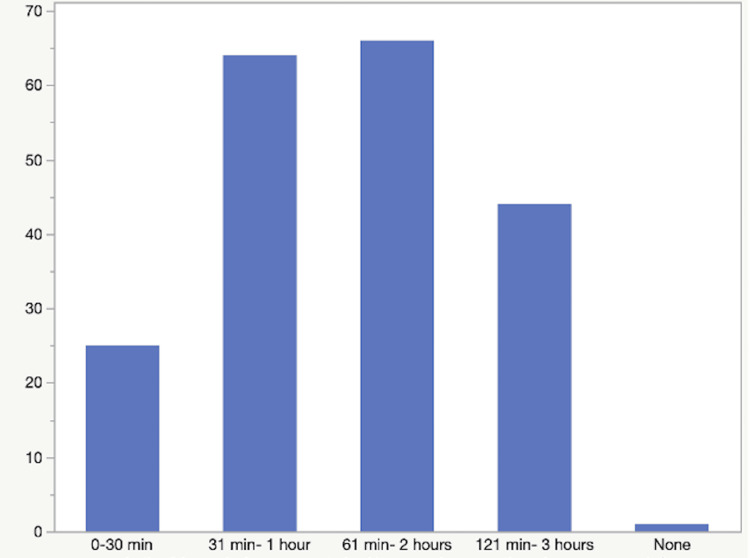
Amount of screen time the respondents engaged in, in the three hours before their bedtime

Figure 2 depicts the number of hours respondents slept on average. The median was eight hours, with an interquartile range of seven to nine hours. Figure 3 depicts the amount of screen time respondents spent in the three hours prior to their bedtime.

Measuring somnolence in the cohort

**Figure 4:**
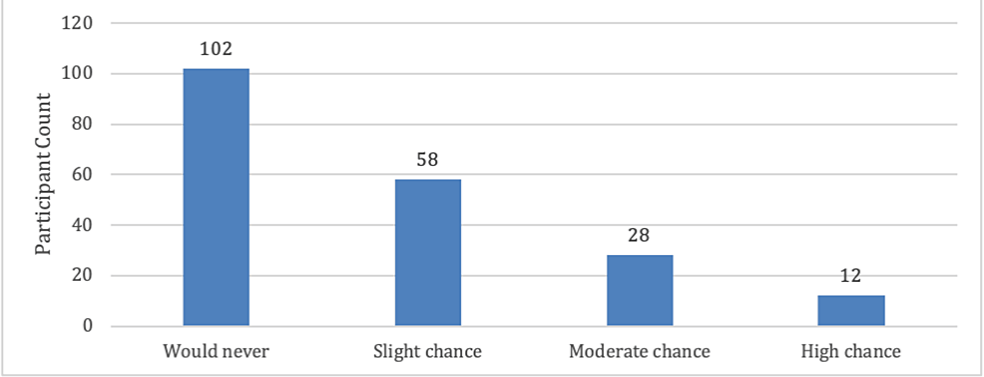
Participants likelihood of falling asleep during inactive periods of day time

In the study, information related to work demands, the number of sleep hours, and symptoms of sleep deprivation was collected to understand the overall sleep quality of the cohort. Coping ability varies with the amount of sleep people experience. Participants were asked to what extent they experienced sleepiness throughout the day while inactive. While more than half (n=102, 51%) of respondents report never falling asleep during daytime periods of inactivity, 58 (29%) report a slight chance, 28 (14%) report a moderate chance, and 12 (6%) report a high chance. These findings are visualized below (Figure 4).

The workplace

**Figure 5:**
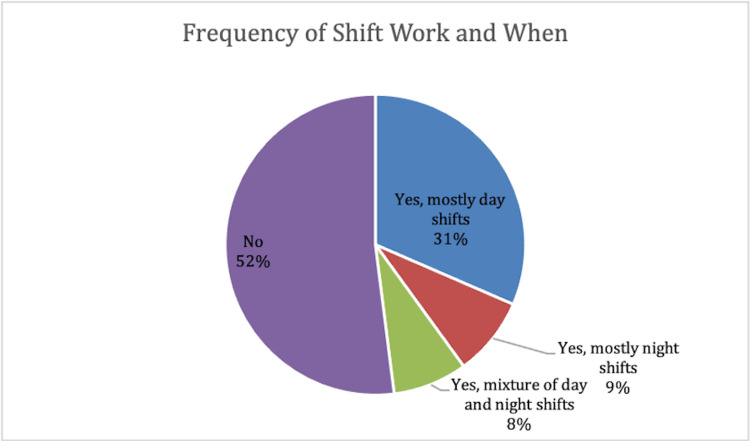
The occurrence and timing of shift work within cohort

As professional obligations play a role in one’s sleep timing and duration, participants were asked about the characteristics of work. Shift work and timing, as well as the number of hours individuals worked per day, were of specific interest. The most common response in the cohort was not participating in shift work by 104 individuals. 63 respondents report mostly daytime shifts, 17 respondents report mostly night shifts, and 16 individuals disclose a mixture of day and night shifts. Findings related to shift work are displayed below (Figure 5).

Along with shift work, participants were asked about the duration of an average workday. While most report a standard eight-hour workday, this represents less than half (n=94, 47%). 61 participants, or 30.5%, work less than 8 hours, 38 (19%) work for 10 hours, and 7 or 3.5% work for 12 hours on average. These results are shown in Table 2.

**Table 2:** Length of workday for participants, on average

Answers	Answers (%)	Count
Less than 8 hours	30.50%	61
8 hours	47.00%	94
10 hours	19.00%	38
12 hours	3.50%	7

Factors related to daytime sleepiness and coping mechanisms

In the survey, participants reported their propensity to fall asleep during the daytime. This urge reflects symptoms of untimely, unwanted sleepiness and is a proxy for the poor quality of sleep. Multivariate analysis was used to take into consideration demographic variables as well as information reported about participants’ occupations to understand sources of daytime sleepiness. Race (p=0.112), gender (p=0.426), age (p=0.063) were among the demographic factors that were not statistically significant in predicting unwanted sleepiness during the day. In addition, shift work/shift timing (p=0.260) and screen time before bed (p=0.785) were not statistically significant for daytime sleepiness. However, the length of participants’ workdays (p=0.001) and their income levels (p=0.008) were found to affect sleep quality. Lengthier workdays and lower income levels were associated with an increased likelihood of falling asleep during inactive periods of the day. While the length of one’s workday and its influence on sleep quality are intuitive, income is seemingly more indirect.

**Figure 6:**
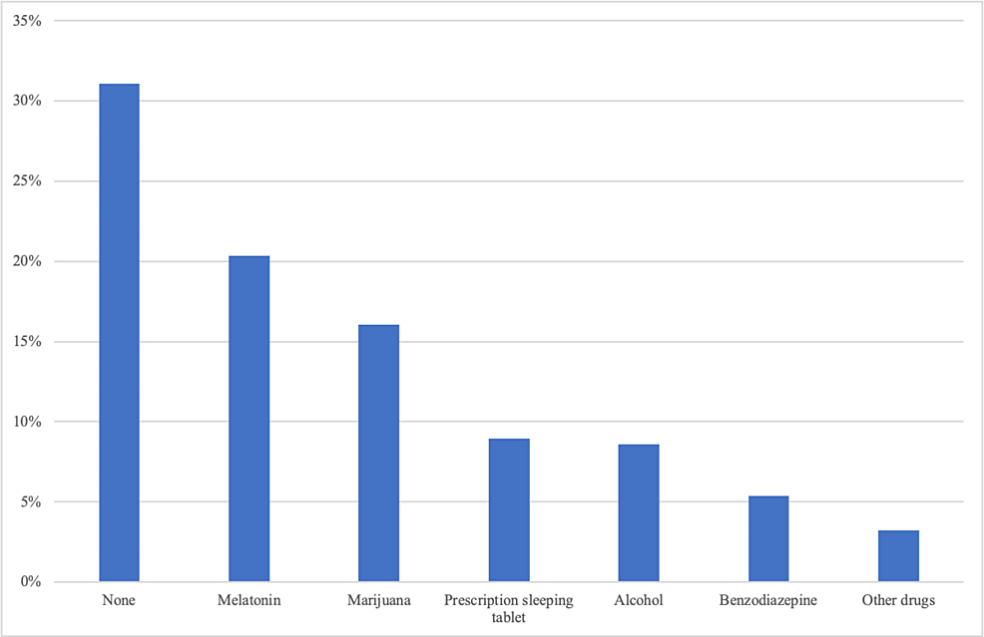
Medications taken by participants as aids for sleep

Participants were also asked about their use of oral medications as sleeping aids. Melatonin was the most used drug by 57 (38.5%) participants, followed by cannabis (45, 22.5%), sleeping tablets (25, 12.5%), alcohol (24, 12%), antihistamines (18, 9%), benzodiazepines such as Xanax® (15, 7.5%), and other drugs (9, 4.5%). These findings are shown in Figure 6.

## Discussion

The findings of this study highlight multiple important correlates of sleep quality. Half of the participants reported earning less than $50,000, and lower income strongly correlated to an increased propensity to fall asleep during the day. Individuals with lower incomes are more likely to face financial stress. Although this relationship is seemingly less straightforward, financial stress has previously been correlated with shorter sleep durations in young adults and sleep disturbances in older adults [11]. Consistently poor nighttime sleep quality would increase the likelihood that individuals fall asleep during the day. Stress in general has been shown to lead to poor sleep quality, and thus it would follow that financial stress would do the same and can lead to excessive daytime sleepiness [12,13]. Individuals with increased financial stress are also more likely to have increased work hours, another factor demonstrated to previously impact sleep and corroborated by this survey [14]. In this study, the length of an individual’s workday had a strong statistical correlation with the likelihood that they fell asleep during the day. This relationship can potentially be explained by longer workdays reducing the amount of time an individual is able to dedicate to sleep, as they may have other responsibilities in addition to work, such as childcare, that necessitate engagement after the workday but before an individual is able to sleep. More than half of respondents indicated having a workday longer than eight hours a day. In general, participants reported a median amount of eight hours of sleep, with the middle 50% ranging between seven and nine hours of sleep. With 25% of participants having lower than the recommended amount of sleep but a much higher percentage reporting excessive daytime sleepiness, factors outside of sleep length are likely affecting sleep quality and having a large impact on the data.

It is notable that screentime before bed, shift work, and shift timing were not correlated to excessive daytime sleepiness. This disagrees with previous studies that demonstrated their impact on sleep quality or length. This could be the result of changes in technology that can filter out blue light, a smaller sample size of 200 individuals, or a relatively few individuals in the study who work night shifts. Additionally, the sample may not be perfectly representative of national data. Only 5% of individuals had insomnia, 6.5% of individuals had obstructive sleep apnea, and 25% of individuals reported sleep disorders, all data that are lower than national averages [5,15-18]. A sample with a higher prevalence of sleep disorders may alter the results. Further research should be conducted in the aforementioned areas. The timing of the survey could also impact the results. The COVID-19 pandemic altered workplace environments for many, including increased remote work or sick leave. Although unclear, the circumstances of the pandemic cannot be ignored in terms of their impact on work environments. The results of the survey also indicate that 41.5% of individuals have mild, moderate, or excessive daytime sleepiness. This could also be impacted by the overall stress or related changes due to the pandemic, as the quality of sleep has been shown to have worsened during the pandemic [19-20]. Excessive daytime sleepiness was also believed to have increased due to the pandemic in certain subgroups, like adolescents and healthcare workers [21-22]. Although much of the United States has returned to policies and activities similar to before the pandemic, the longitudinal impact of the pandemic cannot be underestimated in impacting sleep. Interestingly, 2/3 of the respondents also reported using a sleep aid.

Limitations to the current study are those inherent to most anonymous survey studies and stem from the lack of control the researchers have over the respondents and their answers. Any adult living in the United States could have answered our survey. Their motivations to participate in the survey may have skewed respondents' answers. In an anonymous online setting, the truthfulness of answers is also difficult to ascertain, although there is no incentive to purposefully be deceptive. A total of 200 responses were collected, which represents only 6 × 10-7% of the adult US population. Thus, the data may not be generalizable. Given this, these data should be interpreted as a snapshot in time of an audience that self-selects to answer a survey on sleep quality and possibly a starting point for informing the conversation on sleep habits.

## Conclusions

An overall assessment of the level of daytime sleepiness was obtained via an online survey. This study investigates multiple factors related to sleep and sleep hygiene, including the impact of shift work, screen time, income, and demographics. While online anonymous surveys can be a valuable tool for collecting data, they come with limitations inherent to survey methodology. These include self-selection bias, a lack of control over participants and their environment, and, due to the anonymous nature of the responses, a potential lack of accuracy or authenticity in responses. These factors may limit the generalizability of the current study's results.

## References

[1] (2023). Information about sleep. https://www.ncbi.nlm.nih.gov/books/NBK20359/.

[2] Irwin MR (2015). Why sleep is important for health: a psychoneuroimmunology perspective. Annu Rev Psychol.

[3] Hirshkowitz M, Whiton K, Albert SM (2015). National Sleep Foundation's sleep time duration recommendations: methodology and results summary. Sleep Health.

[4] Shechter A, Kim EW, St-Onge MP, Westwood AJ (2018). Blocking nocturnal blue light for insomnia: a randomized controlled trial. J Psychiatr Res.

[5] Kaur H, Spurling BC, Bollu PC (2023). Chronic Insomnia. https://pubmed.ncbi.nlm.nih.gov/30252392/.

[6] Mahfoud Y, Talih F, Streem D, Budur K (2009). Sleep disorders in substance abusers: how common are they?. Psychiatry (Edgmont).

[7] Savarese M, Di Perri MC (2020). Excessive sleepiness in shift work disorder: a narrative review of the last 5 years. Sleep Breath.

[8] Johns MW (1991). A new method for measuring daytime sleepiness: the Epworth sleepiness scale. Sleep.

[9] Johns MW (1992). Reliability and factor analysis of the Epworth Sleepiness Scale. Sleep.

[10] Johns MW (2000). Sensitivity and specificity of the multiple sleep latency test (MSLT), the maintenance of wakefulness test and the epworth sleepiness scale: failure of the MSLT as a gold standard. J Sleep Res.

[11] Du C, Wang W, Hsiao PY, Ludy MJ, Tucker RM (2021). Insufficient Sleep and Poor Sleep Quality Completely Mediate the Relationship between Financial Stress and Dietary Risk among Higher Education Students. Behav Sci (Basel).

[12] Almojali AI, Almalki SA, Alothman AS, Masuadi EM, Alaqeel MK (2017). The prevalence and association of stress with sleep quality among medical students. J Epidemiol Glob Health.

[13] Åkerstedt T, Orsini N, Petersen H, Axelsson J, Lekander M, Kecklund G (2012). Predicting sleep quality from stress and prior sleep--a study of day-to-day covariation across six weeks. Sleep Med.

[14] Peltz JS, Bodenlos JS, Kingery JN, Rogge RD (2021). The role of financial strain in college students' work hours, sleep, and mental health. J Am Coll Health.

[15] Morin CM, Jarrin DC (2022). Epidemiology of insomnia: prevalence, course, risk factors, and public health burden. Sleep Med Clin.

[16] Petrov ME, Lichstein KL, Baldwin CM (2014). Prevalence of sleep disorders by sex and ethnicity among older adolescents and emerging adults: relations to daytime functioning, working memory and mental health. J Adolesc.

[17] McArdle N, Reynolds AC, Hillman D, Moses E, Maddison K, Melton P, Eastwood P (2022). Prevalence of common sleep disorders in a middle-aged community sample. J Clin Sleep Med.

[18] Madan Jha V (2023). The prevalence of sleep loss and sleep disorders in young and old adults. Aging Brain.

[19] Didriksen M, Werge T, Nissen J (2021). Impact of COVID-19 pandemic on sleep quality, stress level and health-related quality of life-a large prospective cohort study on adult Danes. Int J Environ Res Public Health.

[20] Richter SA, Ferraz-Rodrigues C, Schilling LB, Camargo NF, Nunes ML (2023). Effects of the COVID-19 pandemic on sleep quality in children and adolescents: a systematic review and meta-analysis. J Sleep Res.

[21] da Silva BB, de Melo MC, Studart-Pereira LM (2022). Adolescents' sleep quality during the COVID-19 pandemic. Sleep Sci.

[22] Erdoğan A, Berktaş DT, Öksüz AN, Şahin AR, Koçyiğit BF (2022). The impact of COVID-19 pandemic on sleep quality in healthcare workers in Turkey. Egypt J Neurol Psychiatr Neurosurg.

